# A Soldier’s Life Saved by a Dream

**Published:** 1889-04

**Authors:** 


### A SOLDIER'S LIFE SAVED BY A DREAM.

"A man of the name of Joe Williams had told a dream to his fellow soldiers, some of whom related it to me months previous to the occurrence, which I relate. He dreamed that he crossed a river, marched over a mountain and camped near a church located in a wood, near which a terrible battle ensued, and in a charge just as he crossed a ravine he was shot in the heart. On the ever memorable 7th of December, 1861 (Battle of Prairie Grove, Northern Arkansas), as we moved a double quick to take our place in the line of battle, then already hotly engaged, we passed a church, a small frame building. I was riding in the flank of the command opposite to Williams, as we came in view of the house. 'That is the church I saw in my dream,' said he. I made no reply, and never thought of the matter until evening. We had broken the enemy's lines and were in full pursuit, when we came to a dry ravine in the wood, and Williams said: 'Just on the other side of this ravine I was shot in my dream, and I'll stick my hat under my shirt.' Suiting the action to the word he doubled up his hat as he ran along and crammed it into his bosom. Scarcely had he adjusted it when a minie ball knocked him out of line; jumping up quickly he pulled out his hat, waved it over his head shouting: 'I'm all right.' The ball raised a black spot about the size of a man's hand, just over the heart, and dropped into his shoe."

